# The interplay between selective and nonselective inhibition during single word production

**DOI:** 10.1371/journal.pone.0197313

**Published:** 2018-05-10

**Authors:** Ruben D. Vromans, Suzanne R. Jongman

**Affiliations:** 1 Tilburg center for Cognition and Communication (TiCC), Tilburg University, Tilburg, The Netherlands; 2 Max Planck Institute for Psycholinguistics, Nijmegen, The Netherlands; University of Dundee, UNITED KINGDOM

## Abstract

The present study investigated the interplay between selective inhibition (the ability to suppress specific competing responses) and nonselective inhibition (the ability to suppress any inappropriate response) during single word production. To this end, we combined two well-established research paradigms: the picture-word interference task and the stop-signal task. Selective inhibition was assessed by instructing participants to name target pictures (e.g., dog) in the presence of semantically related (e.g., cat) or unrelated (e.g., window) distractor words. Nonselective inhibition was tested by occasionally presenting a visual stop-signal, indicating that participants should withhold their verbal response. The stop-signal was presented early (250 ms) aimed at interrupting the lexical selection stage, and late (325 ms) to influence the word-encoding stage of the speech production process. We found longer naming latencies for pictures with semantically related distractors than with unrelated distractors (semantic interference effect). The results further showed that, at both delays, stopping latencies (i.e., stop-signal RTs) were prolonged for naming pictures with semantically related distractors compared to pictures with unrelated distractors. Taken together, our findings suggest that selective and nonselective inhibition, at least partly, share a common inhibitory mechanism during different stages of the speech production process.

## Introduction

When we talk to another person, we not only have to think of what to say, but also of what *not* to say. For instance, many irrelevant thoughts may come to mind and need to be suppressed because they are contextually or socially inappropriate. In addition, we do not produce an infinite stream of speech, but instead are able to stop ourselves when we notice that someone else is about to speak. It thus seems that some processes in language production require different forms of inhibition that allow for the emergence of goal-directed speech behavior. The present study focuses on the interplay between selective and nonselective inhibition during single word production. We first discuss the main components of word production and which type of inhibition, selective and nonselective, may be necessary for these production processes. Next, we discuss what is known about the interplay between these two forms of inhibition in the nonverbal domain, which leads to our hypotheses for such an interplay in word production: the interest of our study.

The production of spoken words appears to be a complex cognitive task which entails a number of mental processes that take place between activating an idea and activating the sounds you need to express the idea [1,2]. In the conceptual preparation stage, production starts with a concept that the speaker wishes to communicate. The output of this first process, a lexical concept, leads to the second stage of lexical selection. During this stage, lemmas–mental representations providing syntactic information including gender, tense, or number etc.–that correspond to the lexical concept become activated. During the word-form encoding stage, the appropriate morpho-phonological and phonetic information is encoded, after which a speaker must program a set of motor movements in the articulation stage in order to create sounds that the listener will perceive as speech. Indefrey and Levelt [3,4] estimated that, on average, the amount of time that people need to plan a verbal response in a simple picture-naming task is around 600 ms. Their meta-analysis revealed that conceptual preparation should be completed within 200 ms after picture onset, lexical selection should occur between 200 and 275 ms, and word-form encoding between 275 and 600 ms (with phonological encoding between 275 and 355 ms, syllabification around 355 ms and phonetic encoding around 455 ms). Finally, at around 600 ms, the last and final stage of articulation should start.

Speaking is not only complex but also goal-directed. Indeed, while speaking, it seems necessary to have fine control over our speech acts, utterances, and speech planning processes [5], in order to avoid the production of random words at random moments. Moreover, while speaking, many irrelevant thoughts may come to mind and need to be suppressed, and many words may be activated that we should not express because they are contextually inappropriate [6]. It thus seems that all of these processes in language production require some form of *inhibition*. However, it has been proposed that inhibition is not a unitary construct but can be considered as a set of closely related abilities [7,8,9]. As such, several taxonomies of types of inhibitory control processes have been proposed [8,9]. An important distinction is between *selective* and *nonselective* inhibition (cf. [6,10]).

Selective inhibition refers to the suppression of specific alternative responses that are considered to be strong competitors to a target response [10]. This type of inhibition is typically involved in classic conflict tasks such as the Stroop, Simon or Eriksen flanker tasks where competing responses are activated by distractors (incongruent trials) that need to be selectively inhibited in order to select the correct response. The contribution of selective inhibition in speaking may include the suppression of incorrect names that are co-activated while speaking [6,11,12]. Evidence for the activation of multiple lemmas comes from the picture-word interference task [13] where participants are presented with pictures (e.g., cat) that have words printed on top of them. Some of these distractor words are semantically related to the object in the picture (e.g., dog), and others are unrelated (e.g., house). It is the participants’ task to name the picture out loud as fast and as accurately as possible, while ignoring the distractor word that is superimposed on the picture. A well-established finding in this task is that naming latencies are longer in the semantically related than in the unrelated condition (e.g., [13,14,15]), which is called the *semantic interference effect*.

The locus of the semantic interference effect has become a topic of debate in psycholinguistics, whereby accounts of lexical selection-by-competition and response-exclusion are often contrasted. According to the lexical selection-by-competition account, the effect arises during the lexical selection stage. Semantically related distractors are activated not only by the superimposed word but also by spreading activation from the concept of the related target picture and hence compete more strongly with targets than unrelated distractors [2,14,16]. In this account, selective inhibition is needed in the semantically related condition in order to reduce the competition between the target lemma and the semantically activated competitor [6,12]. According to the response-exclusion account, the semantic interference effect is postlexical. Before articulation, production-ready representations of distractors must be excluded as potential responses from the single-channel output buffer. Unrelated distractors can be excluded faster than related distractors, because they satisfy fewer semantic constraints demanded by the target picture. To take the previous example, when naming a picture of a cat it is easier to remove the distractor house from the output buffer than the distractor dog, as it does not satisfy the response-relevant criteria of naming an animal. In the response-exclusion account, the time required to select the target lemma does not depend on the activation of other lemmas and selective inhibition is therefore not required [17,18].

The other form of inhibition, nonselective inhibition, refers to the suppression of any response that is considered to be incorrect or inappropriate. This type of inhibition is considered to be nonselective, because it is needed to suppress the execution of *any* planned motor responses. Nonselective inhibition is typically involved in tasks such as the stop-signal task [19,20], where participants are presented with a continuous series of stimuli and are asked to judge as fast and accurately as possible whether they see stimulus *X* or stimulus *Y* on the screen by pressing the corresponding response buttons. However, on a subset of stop-trials, the go-stimulus is–after a fixed or variable stop-signal delay (SSD)–followed by a stop-signal (i.e., an auditory beep or a visual stimulus), indicating that participants should withhold their response to the go stimulus. Here, nonselective inhibition is indexed by the amount of time that is needed to withhold a response when a stop-signal is given. The latency of the stop-process cannot be observed directly, as a successful stop-trial results in no response. To infer the latency of the stop-process the independent horse-race model is used [19,20], which describes nonselective inhibition performance as a race between a go process (activated by the go-stimulus) and a stop process (activated by the stop-signal). It is assumed that on half of the stop-trials the participant is able to successfully inhibit the motor response, and by using the distribution of go-RTs the *stop-signal reaction time* can be inferred (henceforth stop-signal RT, for more details see Method section). The stop-signal RT is a validated measure of nonselective inhibition (for a review, see [21]). In terms of stopping performance, longer stop-signal RTs reflect general slowing of inhibitory processes and thus indicate a lower level of nonselective inhibitory performance.

The contribution of nonselective inhibition in speech production may include the suppression of an already initiated speech act. For instance, when a speaker has the intention to say something but notices that the communication partner is still talking, a speaker must withhold a planned verbal responses before it is his/her turn to speak [22]. A few studies have demonstrated that nonselective inhibitory control performance (assessed by a vocal stop-signal task) over verbal responses is comparable with inhibitory control over manual responses [23–28].

Previous correlational research suggests that selective and nonselective inhibition are distinct processes in language production (e.g., [6]), by showing that nonselective inhibition (as measured by stop-signal RTs) does not correlate with selective inhibition (as measured by the semantic interference effect). However, these results were obtained by using two separate inhibition tasks and a lack of a correlation does not prove complete independence, leaving open the possibility that both types of inhibition may in part rely on the same inhibitory mechanisms [20]. Until now, support for a functional relationship between selective and nonselective inhibition comes from the nonverbal domain, where different inhibitory paradigms requiring binary hand movements have been combined. For instance, the stop-signal task has been combined with classic conflict tasks that measure selective inhibition such as the arrow version of the Eriksen flanker task [29], but also the manual version of the Stroop task [30,31]. These studies showed that nonselective inhibition was impaired by the use of selective inhibition as revealed by prolonged stop-signal RTs on incongruent trials, suggesting that both types of inhibition may rely on the same inhibitory mechanism [30,31]. However, very little attention has been paid to a possible interference between different inhibitory control mechanisms during speech production. It therefore remains an open question whether the observed interplay between selective and nonselective inhibition in the nonverbal domain generalizes to the verbal domain.

The main aim of the present study was to determine whether selective inhibition influences nonselective inhibition during single word production. An empirical way of clarifying the interplay between different inhibitory functions is by combining different inhibitory paradigms (cf. [29,31,32]). To this end, we combined two well-established research paradigms: the picture-word interference task and the stop-signal task. More specifically, we used the type of distractor (semantically related vs. unrelated) as an experimental factor for testing selective inhibition, and presented a visual stop-signal after a fixed delay to assess nonselective inhibition. Furthermore, we tried to investigate whether this interplay between the two types of inhibition would change dependent on which word production stage had been reached. Based on the time estimates provided by Indefrey and Levelt [3,4], we tested two fixed SSDs in order to induce a conflict after 250 ms (aimed at tapping into the lexical selection stage) and after 325 ms (aimed at tapping into the word-encoding stage). These delays also correspond to average delays observed in previous studies that used a verbal stop-signal task [27] and in combination with Transcranial Magnetic Stimulation [23].

Based on the findings reported above (e.g., [6,13,14]), we expected to replicate the semantic interference effect, by obtaining longer naming latencies for pictures with semantically related distractors compared to pictures with unrelated distractors on go-trials (i.e., where no stop-signal is presented). Given that nonselective inhibition in the stop-signal tasks interferes with the suppression of responses activated by irrelevant competing information–at least in the nonverbal domain [29,31,32]–we expected that this interference generalizes to the verbal domain as well, under the assumption that selective inhibition is required in word production. Thus, stop-trials where selective inhibition is applied capture more inhibitory resources, which means that there are less processing resources available for nonselective inhibition. Hence, we expected stop-signal RTs to be increased for pictures with semantically related distractors compared to unrelated distractors, indicating harder nonselective inhibitory control to pictures with semantically related distractors.

We tested whether the effect of selective inhibition on nonselective inhibition would be a specific effect relating to whether a lemma has been selected or not. According to the lexical selection-by-competition account, only the lexical selection stage requires selective inhibition and not the word-encoding stage. For the first stage an interaction with nonselective inhibition is expected but not for the second stage. If our stop-signal delays of 250 and 325 ms target lexical selection and word-encoding respectively, we expect an interaction between selective and nonselective inhibition at SSD 250 only. These predictions would follow only from accounts of word production that postulate lexical selection to be a competitive process requiring selective inhibition. Accounts where no such inhibition is required would not predict any interplay between selective and nonselective inhibition. The semantic interference effect and a main effect of nonselective inhibition should still be found, but stop-signal RTs should be identical for pictures with related and unrelated distractors for both SSDs.

## Method

### Participants

A group of 37 undergraduate or postgraduate students (11 men, *M*_age_ = 21.41 years (*SD* = 1.82), range: 18 to 26 years) participated in the study. They were recruited from the participant pool of the Max Planck Institute for Psycholinguistics, Nijmegen. All participants reported to be native speakers of Dutch, to be right handed, to have no dyslexia or other speech or language impairments, no hearing problems, no color-blindness, and to have normal or corrected-to-normal vision. They gave written informed consent and received 8 euros for their participation. Ethical approval was granted by the Ethics Board of the Faculty of Social Sciences of the Radboud University Nijmegen.

### Materials and design

Experimental stimuli consisted of 40 line drawings of common objects selected from a stimulus database of normed pictures [33]. In addition, there were four practice pictures that were not part of the stimulus set. All picture names were monosyllabic and from different semantic categories. Based on the norming study by Severens et al. [33], the average log word-form frequency was 1.72/million (*SD* = 0.41), the average name agreement was 93 percent, and the average age of acquisition was 4.9 years (*SD* = 1.1 years). The pictures fitted to a virtual frame of 8 × 8 cm on the computer screen and were shown as black line drawings on a white background in the center of the screen.

**Fig 1:**
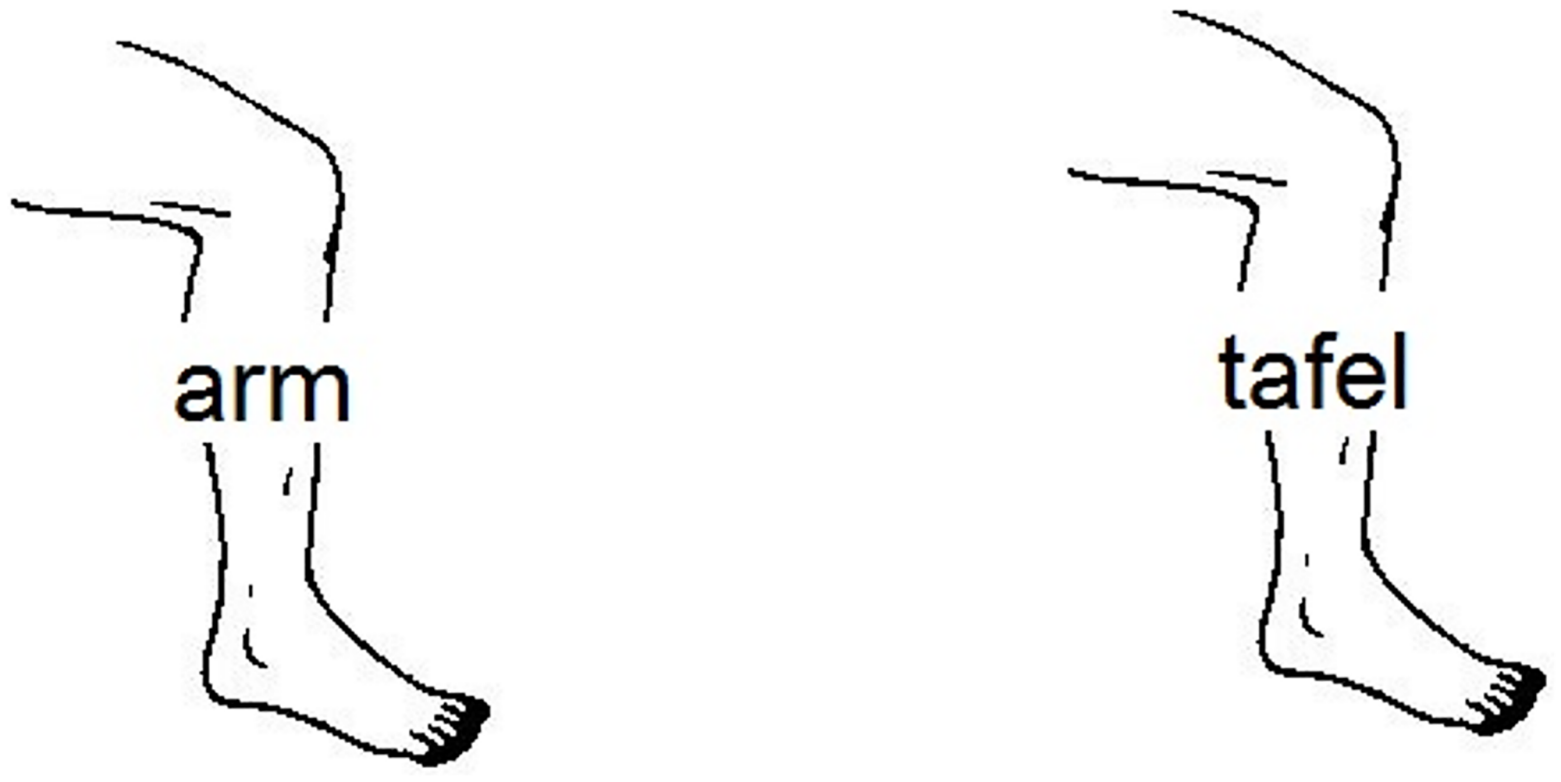
Example stimuli for the semantically related (left) and unrelated (right) conditions (target: been [leg]; distractors: arm [arm], tafel [table]).

In the semantically related condition, the pictures were combined with written distractor words from the same semantic category. For instance, the target ‘leg’ was combined with the related distractor word ‘arm’. In the unrelated condition, the same pictures and distractor words were used, but here the distractors were combined with targets from a different semantic category. For instance, the target ‘leg’ was combined with the unrelated distractor word ‘table’ (see S1 Table for the complete set of pictures with their distractors). The distractors were superimposed in the center of the pictures and were presented in black, lower case Arial font of 26-point size. Two example stimuli are shown in Fig 1.

In total, the task consisted of 320 trials, distributed across four test blocks of 80 trials each. Each block contained the complete stimulus set, that is, the 40 target pictures combined with both semantically related and unrelated distractors. This stimulus set was the same for all four blocks. In addition, each test block contained 60 go-trials (30 related vs. 30 unrelated) and 20 stop-trials (10 related vs. 10 unrelated) in which the two stop-signal delays (SSD250 and SSD325) occurred equally often. The test blocks only differed in terms of the combinations of go-and stop-trials with the related and unrelated distractor items. Over the four blocks, the 40 stop-trials in SSD250 consisted of 20 pictures presented both in the related and unrelated condition and the 40 stop-trials in the SSD325 consisted of the other 20 target pictures in each condition. Which target pictures occurred in SSD250 or SSD325 was counterbalanced across participants. For each participant, the order of trials within each block was pseudorandomized using the program Mix [34], such that no more than four target pictures from the same condition appeared in succession, and that participants never named two pictures that were semantically related or started with the same phoneme in a row.

### Procedure

The participants were tested individually. The experiment took place in a dimly-lit sound-proof booth at a comfortable viewing distance in front of a monitor. Before entering the booth, participants received a written explanation of the project, and gave written informed consent. They also filled out a short sociodemographic questionnaire. All participants first completed the picture-word interference stop-signal task, and subsequently performed another short experimental task. The second experimental task was not relevant for the present study, and, hence, the corresponding data were not reported here. This task was always completed after the picture-word interference stop-signal task, and therefore could not have influenced the results. After the experimental session, participants were debriefed and paid for their participation. An experimental session lasted about 45 minutes per participant.

At the beginning of the task, participants were familiarized with the set of pictures by viewing each picture and its name on the screen. They were instructed to use only these picture names. Whenever they had understood the picture and its name, they could press a button to continue to the next picture. After the familiarization phase, a practice phase was administered consisting of 8 trials (4 related vs. 4 unrelated distractors; each condition containing 3 go-trials and 1 stop-trial). During both the familiarization and practice phase, the experimenter was physically present in the booth in order to correct errors made by the participant. The practice phase was followed by the experimental phase, including four test blocks separated by short breaks. Before the experimental phase, it was made clear to the participants that stopping and going were equally important and that it would not always be possible to stop their vocal responses. In addition, they were clearly instructed not to slow down their responses over the course of the experiment. Each test block was preceded by an instruction screen with a reminder to the participant: “Once again, do not WAIT for the stop-signal to occur, but respond as FAST as you can”.

**Fig 2:**
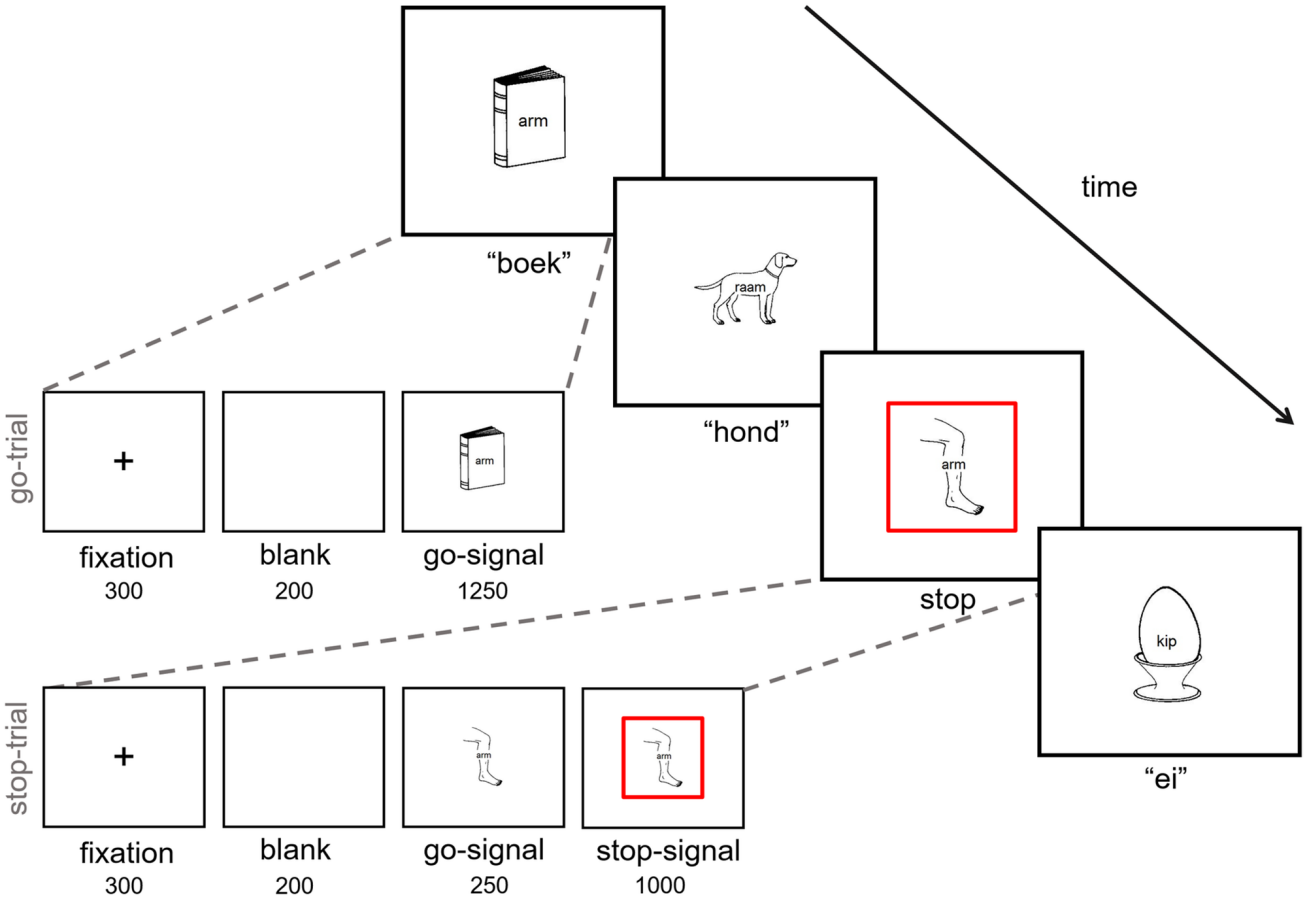
Depiction of a trial course in the picture-word interference stop-signal task.

On each trial of the test blocks, a fixation cross was presented for 300 ms in the middle of the screen, followed by a blank screen for 200 ms. Then, a picture with a distractor word appeared at the middle of the screen for a maximum of 1250 ms and participants were instructed to name the object in the picture out loud, as fast and as accurately as possible. They were also explicitly instructed to name only the picture and not the word that was printed on it. On 25% of the trials, a visual stop-signal (a red square frame (0.80 cm) surrounding the picture border) was presented after a variable delay indicating that participants should not say anything. The stop-signal delay (SSD; the stimulus onset asynchrony between the picture-onset and the visual stop-signal-onset) was either set at 250 ms or 325 ms, respectively. Both SSDs occurred equally often. The inter-trial interval was 2000 ms and was independent of naming latencies. An experimental session of the task took about 25 minutes per participant. A depiction of a trial course is shown in Fig 2.

### Data analyses

Analyses were performed on six variables. For the go-trials we analyzed naming latencies (Go RT), missed responses, and incorrect responses. Naming latencies were determined manually using the speech analyses program Praat [35]. Missed responses on go-trials were defined as cases where the participant did not produce any verbal response. Errors on go-trials were based on two types: incorrect responses and disfluencies. Incorrect responses were defined as cases where the participant used picture names that differed from those given in the familiarization phase. Disfluencies were defined as cases where the participant repaired the utterance, stuttered, or started with a filler word (e.g., “uh”). Errors and missed responses on go-trials were excluded from the RT analyses. Moreover, for each participant, we removed Go RTs below or above 2.5SD away from the mean for each distractor condition separately. For stop-trials we analyzed failed inhibitions, and the naming latencies for these failed stop-trials. Failed inhibitions were defined as cases where the participant failed to withhold the verbal response. Finally, for each participant the stop-signal RT was calculated for each SSD and distractor type separately.

**Fig 3:**
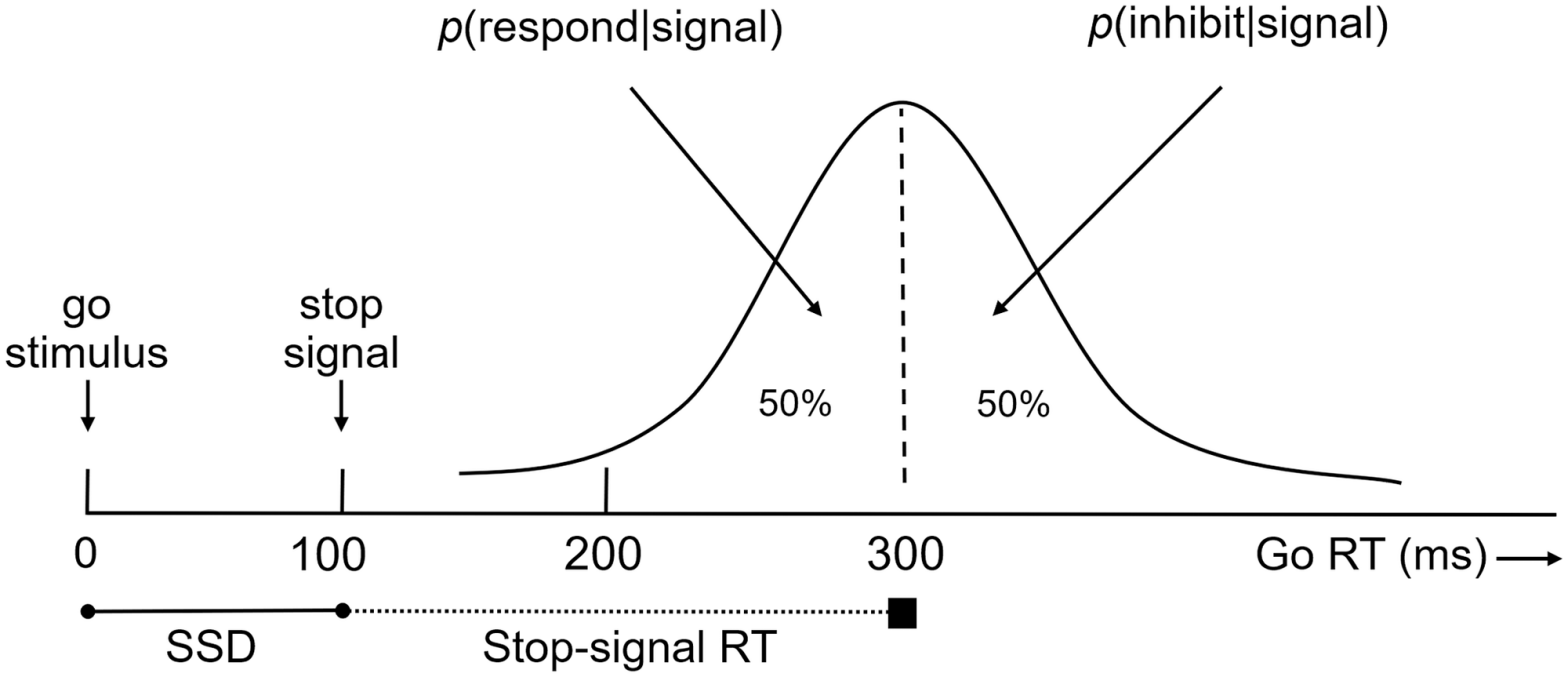
Graphic representation of the independent horse-race model (Adapted from [19]).

Stop-signal RTs were estimated based on the independent horse-race model [19,21]. A graphic depiction of this model is depicted in Fig 3. According to this model, nonselective inhibition performance is described as a race between a go process triggered by a go stimulus and a stop process triggered by a stop-signal. In cases where the go-process finishes before the stop process, the response is executed; in cases where the stop-process finishes before the go-process, the response is inhibited. The curve in the figure shows the distribution of reaction times on go-trials (i.e., trials on which a stop-signal is not presented), representing the finishing time of the response processes. For calculating the stop-signal RTs, we used the integration method–instead of the mean method (see [36])–which allows for a reliable estimation of stop-signal RTs when using fixed delays, as is the case in our experimental task. According to this method, after rank ordering the reaction times on go-trials, the left part of this go RT-distribution is assumed to correspond to the distribution of go RTs on stop-trials on which the participant failed to inhibit the verbal response. The finishing time of the stop process corresponds to the *n*th go RT on go-trials, where *n* is the result of multiplying the total number of go-trials by the probability of responding when a stop-signal is presented [*p*(*respond* | *signal*)], given a particular SSD. For example, on the basis of the numbers in Fig 3, stop-signal RT (200 ms) can be estimated by subtracting SSD (100 ms) from the Go RT marking the 50th percentile (here 300 ms) (for more details about the independent horse-race model, see [19–21]). Thus, the stop-signal RT was calculated as follows: ‘stop-signal RT = *n*th go RT–SSD’.

Data were analyzed using R [37]. Specifically, the *lme4* package [38] was used to fit the linear mixed effects models. For naming latencies (both go-trials and stop-trials) and stop-signal RTs, whether a factor made a significant contribution was determined in two steps. First, models with and without a factor were compared using a likelihood ratio test using the anova function. Factors were dropped that did not reliably contribute to model fit. Second, for the best-fitting model, factors with absolute values of *t* > 2 were considered to significantly contribute to explaining the dependent variable [39]. For errors, logit mixed models were conducted [40]. For logit mixed models, *p*-values are provided for all entered factors and used to determine whether a factor made a significant contribution or not. The maximal random structure was used for each model, unless it failed to converge [41]. The model structure for all models of the go-trials (naming latencies, missed responses, incorrect responses) was identical. Factors were mean-centered. The three models included distractor (related vs. unrelated) as a fixed effect and participant, picture and word as random effects (intercept and slope). The models for stop-trials, thus for failed inhibitions and RTs for these failures, included SSD (250 vs. 325) and distractor as fixed effects as well as their interaction. Participant, picture and word were added as random effects. For the error model the random slope for SSD was added only for participant, as other models did not converge. The RT model included all intercepts and slopes, and the fixed effect SSD was centered because the two levels differed in the number of observations contributing to the analysis.

Naming latencies on go-trials were compared to naming latencies on failed inhibitions on stop-trials, with a model that included trial type, and participant, picture and word as random effects (intercept and slopes). Finally, the model for stop-signal RTs included SSD and distractor and their interaction as fixed effects, and participant as a random effect (intercept only).

### Apparatus

The experimental task was administered on an HP Z400. The monitor of this computer was 16 inch with a resolution of 1280 × 1024, and the operating system was Windows 7 Enterprise. The Presentation^®^ software package (Version 16.5, www.neurobs.com) was used to control the experimental task. Picture naming RTs were recorded online using a Sennheiser microphone and a voice key (which measures voice onset latencies) but were later checked and manually corrected by using the speech analyses program Praat [35].

## Results

The data obtained from one participant were lost due to technical problems with the microphone. Therefore, the data from this participant were excluded from all statistical analyses.

### Analysis of go-trials

**Fig 4:**
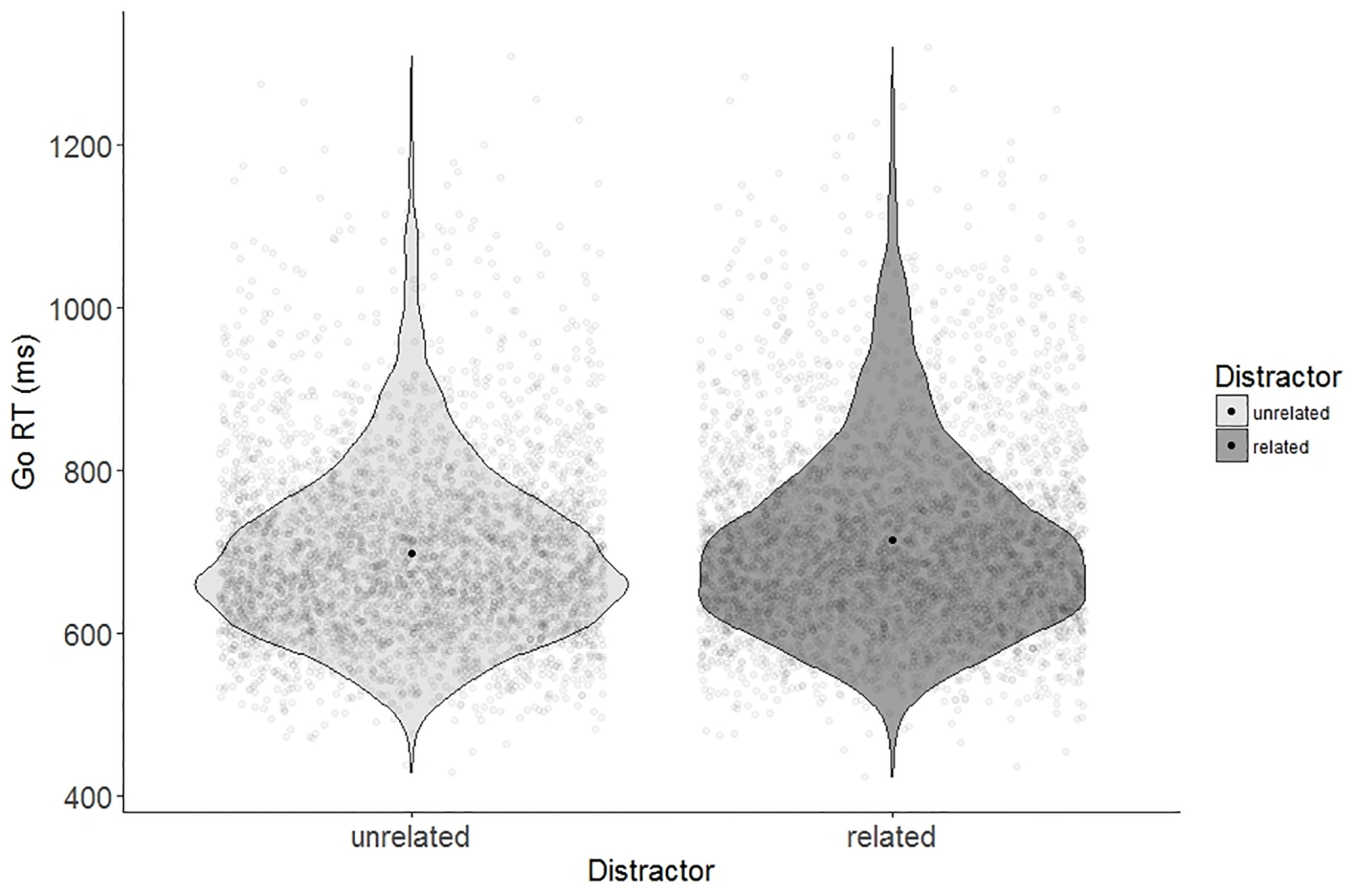
Violin plots of naming latencies go-trials (Go RT), separate for the related and unrelated picture-word combination. Dark dot indicates the mean, light dots are individual data points.

Table 1 shows the mean reaction times on go-trials (Go RTs), proportion of error rates and missed responses on go-trials in the semantically related and unrelated conditions. The best-fitting linear mixed effects model for correct naming latencies included the main effect of distractor (*ß* = 15.56, *SE* = 4.63, *t* = 3.36). Removing distractor significantly decreased model fit (*χ*^*2*^(1) = 10.05, *p* < .01). As expected, the participants’ responses were slower, by 15 ms, in the related (*M* = 715, *SD* = 118) than in the unrelated condition (*M* = 700, *SD* = 109). See Fig 4 for violin plots.

**Table 1:** Mean naming RTs, error rates and missed responses on go-trials per condition.

	Condition			
	Unrelated		Related	
	*M*	*SD*	*M*	*SD*
Go RT* (ms)	700	109	715	118
Errors (%)	2.80	2.62	2.80	2.72
Misses (%)	0.49	0.81	0.79	0.95

*Go RT = reaction times on go-trials

The logit mixed models for errors did not reveal a significant distractor effect. Missed responses occurred in 0.5% in the unrelated condition and 0.8% in the related condition (range 0–3.3%, distractor effect: *ß* = 0.82, *SE* = 0.56, *z* = 1.46, *p =* .15). Incorrect responses were given on 2.8% of trials for both distractor conditions (range 0–10.8%, *ß* = 0.15, *SE* = 0.20, *z* = 0.73, *p* = .47).

### Analysis of stop-trials

**Fig 5:**
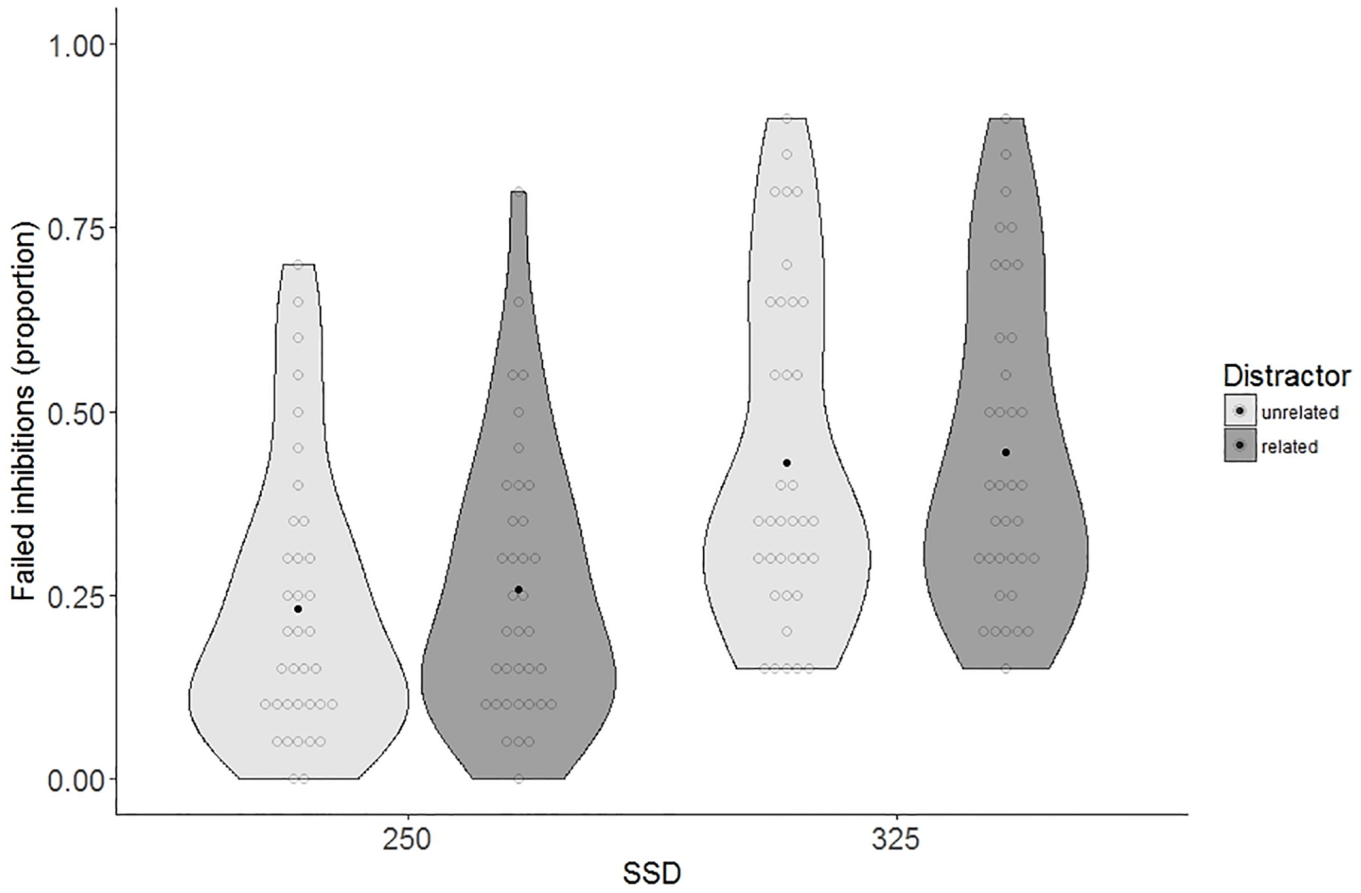
Violin plots of the proportion of failed inhibitions calculated for each participant, separate for SSD and distractor type. Dark dot indicates the mean, light dots are individual data points.

Table 2 displays the proportion of failed inhibition and the naming latencies on stop-trials as a function of SSD and distractor type. Participants failed to inhibit their responses on 34.1% of the stop-trials (see Fig 5). The fixed effect of SSD was significant, with more failed inhibitions occurring for the late SSD compared to the early SSD (43.8% versus 24.5%, *ß* = 1.05, *SE* = 0.14, *z* = 7.65, *p <* .001). The effect of distractor was not significant, neither was the interaction with SSD (*ß* = 0.18, *SE* = 0.14, *z* = 1.30, *p* = .20 and *ß* = 0.11, *SE* = 0.18, *z* = 0.60, *p* = .55, respectively).

**Table 2:** Stop-signal RT, proportion of failed inhibition and failed naming RTs on stop-trials per condition.

	Stop-Signal Delay							
	SSD 250				SSD 325			
	Unrelated		Related		Unrelated		Related	
	*M*	*SD*	*M*	*SD*	*M*	*SD*	*M*	*SD*
Stop-signal RT* (ms)	378	61	398	61	345	52	368	65
Failed inhibitions (%)	23.2	18.9	25.8	18.8	43.1	22.7	44.4	21.2
Failed RT** (ms)	650	151	637	165	635	140	636	150

*Stop-signal RT = stop-signal reaction time

**Failed RT = reaction times on failed inhibition trials

For failed inhibitions, we analyzed the naming latencies. These mistakenly pronounced responses were spoken with an average latency of 638 ms. The best-fitting model was the null model. First dropping the interaction did not result in worse model fit (*χ*^*2*^(1) = 0.25, *p* = .62). Then dropping distractor and SSD did not either (*χ*^*2*^(1) = 0.52, *p* = .47 and *χ*^*2*^(1) = 0.69, *p* = .41, respectively). The naming latencies on these stop-trials (incorrect) were shorter than the previously reported naming latencies on the go-trials (correct). This was formally tested and trial type was indeed a significant predictor of latencies (*ß* = 88.30, *SE* = 14.41, *t* = 6.13). Removing this factor resulted in worse model fit (*χ*^*2*^(1) = 28.42, *p* < .001).

### Analysis of stop-signal reaction times

**Fig 6:**
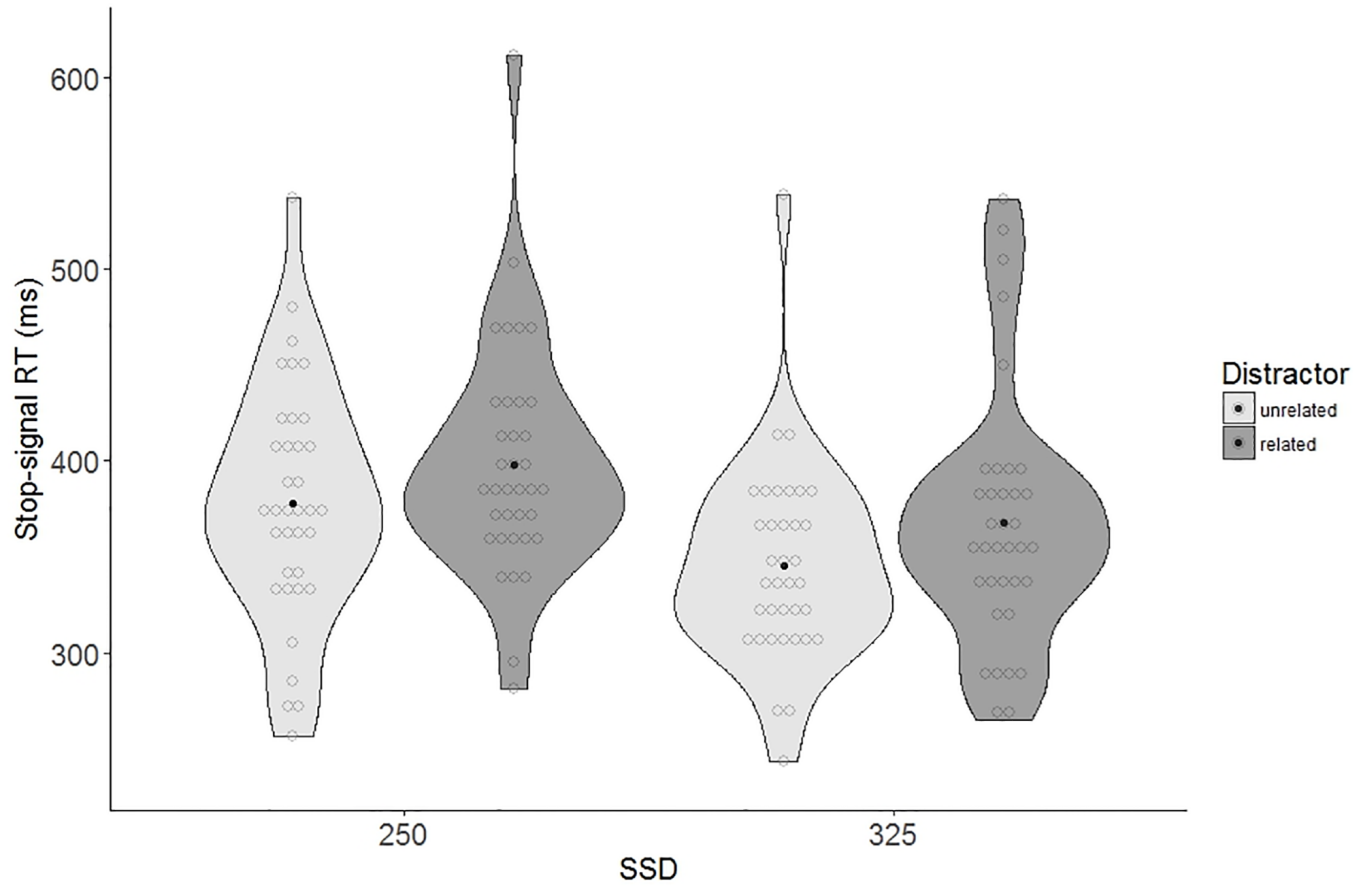
Violin plots of stop-signal RTs, separate for SSD and distractor type. Dark dot indicates the mean, light dots are individual data points.

Table 2 also displays the mean stop-signal RTs on stop-trials as a function of SSD and distractor type. The best-fitting model for stop-signal RTs included both fixed effects of SSD and distractor but not the interaction. Dropping the interaction did not affect model fit (*χ*^*2*^(1) = 0.05, *p* = .83). SSD significantly contributed to the model (*χ*^*2*^(1) = 37.93, *p* < .001), with longer stop-signal RTs for SSD250 compared to SSD325 (SSD250: 388 ms, *SD* = 58 versus SSD325: 357 ms, *SD* = 54; *ß* = -31.51, *SE* = 4.72, *t* = -6.68). Distractor type also significantly contributed to the model (*χ*^*2*^(1) = 19.20, *p* < .001). Stop-signal RTs for unrelated trials were shorter (*M* = 362 ms, *SD* = 54) than for related trials (*M* = 383 ms, *SD* = 60; *ß* = 21.43, *SE* = 4.72, *t* = 4.54). See Fig 6 for the stop-signal RTs as a function of SSD and distractor type.

### Analysis subset fast namers

We selected the participants who on average responded below 700 ms, seventeen individuals in total. These were the fastest responders, and correspond closest to Levelt and Indefrey’s [3,4] estimate of 600 ms average naming speed (but note that the average naming latency of these fast namers was still 647 ms). We can be somewhat more certain that the SSD of 250 ms targeted the lexical selection stage (between 200 and 275 ms when the naming latency is 600 ms), and that the SSD for 325 ms targeted phonological encoding (between 275 and 355 ms) [3,4]. All effects that were significant for the whole group remained significant, and all factors that did not show an effect still did not. Importantly, the interaction between SSD and distractor was still not significant, for neither the proportion of failed inhibitions (*ß* = 0.29, *SE* = 0.27, *z* = 1.11, *p* = .27) or for the stop-signal RTs (*ß* = 8.90, *SE* = 14.28, *t* = 0.62; the model with the interaction did not provide a better fit than the model without the interaction, *χ*^*2*^(1) = 0.41, *p* = .52).

## Discussion

It has become increasingly appreciated that different types of inhibitory control play an important role in the speech production process [6,42]. The goal of the present study was to examine the interplay between selective and nonselective inhibition during single word production. By combining two well-established research paradigms–the picture-word interference task and the stop-signal task–we directly examined whether processes involving selective inhibition could affect processes involving nonselective inhibition. Selective inhibition was assessed by instructing participants to name target pictures in the presence of semantically related or unrelated distractor words; whereas nonselective inhibition was assessed by occasionally presenting a visual stop-signal (on 25 percent of the trials), indicating that participants should inhibit their vocal response. In addition, by experimentally varying the onset of the stop-signal (based on the time estimates provided by Indefrey and Levelt [3,4]), we tried to test whether the interplay changed dependent on which speech production stage had been reached.

The present study yielded several findings. First of all, we replicated the semantic interference effect found in several earlier studies (e.g., [6,12–15]). On go-trials (i.e., trials without a stop-signal), participants needed more time to name the pictures with semantically related distractors than with unrelated distractors words. This finding corroborates previous work by showing that semantic interference in picture naming can also be obtained when participants simultaneously perform the stop-signal task [43]. Similar findings (e.g., Stroop-interference effect in a manual stop-signal task) have also been observed in the nonverbal domain [30,31].

A second finding was that selective inhibition influences nonselective inhibition during single word production. On stop-trials, stopping latencies (i.e., stop-signal RTs) increased for pictures with semantically related distractors compared to unrelated distractors, indicating increased impaired nonselective inhibition to pictures with semantically related distractors. This suggests that trials in the semantically related condition capture more inhibitory resources relative to trials in the unrelated condition, providing evidence for the requirement of inhibition during word production. Our findings are problematic for accounts of word production that argue against a competitive nature of word production [17,18]. Instead our results corroborate the lexical selection-by-competition account that postulates selective inhibition is applied in order to reduce the interference from strongly activated semantic competitors [14]. As a consequence, less inhibitory processing resources are available for nonselective inhibitory control abilities. Collectively, this interplay between selective and nonselective inhibition during speech production means that both types of inhibition rely, in part, on the same inhibitory mechanism (see [29,31,32] for similar reasoning).

Finally, the effect of selective inhibition on nonselective inhibition did not depend on the exact time window in which the two forms of inhibition come into conflict. We found that at both stop-signal delays (SSD250 and SSD325), the stopping latencies were longer for pictures with semantically related distractors than with unrelated distractors. This might suggest that the influence of selective inhibition on nonselective inhibition reflects a general effect (i.e., due to increased inhibitory processing capacity) rather than a specific effect (i.e., due to whether the effect occurred at the lexical selection or word-encoding stage). This is in contrast to our predictions, as we had expected the interaction between selective and nonselective inhibition to be especially evident for the short delay (lexical selection stage) where selective inhibition is required to select the correct object name, and not for the late delay (word encoding stage) where selective inhibition should already have been resolved.

A possible explanation for the non-interaction effect between the onset of the stop-signal and the type of distractor on stopping latencies could be that not only the short SSD tapped into the lexical selection stage but so did the late SSD. Our two SSDs were based both on the time estimates provided by Indefrey and Levelt [3,4] as well as on average SSDs obtained by other studies that used a verbal stop-signal task [23,25,27], which, in turn, were based on average naming latencies of around 600 ms. However, the naming latencies of the present study were substantially longer (around 700 ms), which could mean that both SSDs fell into the time window of lexical selection, and no SSD targeted the word encoding stage. We performed analyses for the fastest namers only, but the interaction between SSD and distractor type still did not reach significance. However, even our fastest namers had an average naming latency of approximately 650 ms, so we cannot be certain that our chosen SSDs really targeted the word production stages we had intended even for this fast subset of participants.

One could argue that we should have selected a later SSD to target word-form encoding, but we were limited to the assumptions of the independent horse-race model that was used to calculate the stop-signal RTs [19]. That is to say, if the stop-signal comes too late, it would be impossible to correctly use nonselective inhibition for any participant, regardless of whether selective inhibition has been applied or not. In terms of the horse-race model, this would imply that the go process would always finish before the stop process, resulting in unreliable estimates of stop-signal RTs. Already with our SSD of 325 ms participants could not inhibit their responses on half of the trials, this number would increase as the delay time increases. As such, we were unable to determine whether the interplay between selective and nonselective inhibition is also involved during later stages of word production.

As we were interested in targeting two different levels of word production, we decided to use fixed SSDs. Nevertheless, we admit that this method has several disadvantages. For instance, the fixed-method approach appears to be sensitive to a speed-accuracy trade-off, where it is more likely that participants can use a response strategy aimed at waiting for the stop-signal to occur in the service of accurate inhibition. Although we attempted to reduce gradual slowing of naming latencies by providing clear instructions before the task and after every block (e.g., [36,44]), the proportion of failed inhibition on stop-trials (34 percent) deviated from the normal proportion obtained in the stop-signal literature (50 percent).

One way to improve the current design is to use a dynamic tracking procedure (i.e., adjusting SSDs after every trial depending on the participant’s performance, also known as the *one-up one down procedure* [45]). Typically, a dynamic tracking procedure of a classic stop-signal task is set up as follows: in case of successful inhibition, the SSD will increase with intervals of 50 ms from the default delay; case of unsuccessful inhibition, the SSD will decrease with the same interval length. A tracking procedure would exclude the possibility for response strategies in the picture-word interference stop-signal task. However, a disadvantage of this tracking procedure is that speech onset latencies are required to be measured by means of voice-keys that can automatically register the onset of speech responses. Most voice-keys have poor accuracy and may not reliably detect the onset of speech responses [46]. As such, researchers prefer to record the vocal responses and determine the speech onset latencies afterwards as was done in the present study (cf. [47]). Therefore, an alternative approach for future research could be to use individually tailored fixed SSDs by adjusting the SSDs to the average naming latencies obtained from a separate picture-naming task ([48], for similar reasoning). By doing so, the fixed method can still be used, the voice-key can be avoided, and a speed-accuracy trade-off is less likely to occur.

A limitation of the present study was that the picture-word interference stop-signal task was restricted to the production of single words. This was mostly done in order to exclude the number of syllables as a possible confounding factor for stopping performance. However, there is reason to assume to stopping speech in the stop-signal paradigm is different from stopping speech in real-life [49]. For instance, naturalistic communicative situations often comprise continuous streams of speech including phrases and sentences, whereas in the present stop-signal task participants only needed to produce one single word on each trial. Interestingly, recent psycholinguistic work suggests that both selective and nonselective inhibition are also involved in more complex forms of language production such as generating noun-phrases (e.g., short phrase: “the fork”, long phrase: “the green fork”) [50]. For future research, it would therefore be interesting to extend these findings to testing interactions between selective and nonselective inhibition during the production of more complex speech like phrases or whole sentences.

In a similar vein, it would be interesting to see what happens if the stop-signal was more like a stop-signal that would occur in naturalistic communication, such as a spoken word. A speaker should stop talking when his or her interlocutor vocally indicates he/she wants the turn to speak. Previous research has shown that combining two tasks that are both linguistic in nature (picture naming and syllable categorization) is more difficult than combining a linguistic and nonlinguistic task (picture naming and tone categorization) [51]. Therefore it might be more difficult to stop speaking in the context of speech than sounds (i.e., a word versus an alarm).

Even with these limitations, we believe the findings from the present study have several important theoretical implications. First of all, the present study adds to a growing body of research that shows stop-signal RTs are similar for inhibiting speech and inhibiting manual responses [24–28] and provide additional evidence for a functional relationship between nonselective inhibition and selective inhibition that can also be found in the verbal domain. This study also shows that the stop-signal task can be combined with the picture-word interference task in order to assess the interactions between different inhibitory functions during speech production [52]. From a methodological stance, this is encouraging for future work aimed at the development of experimental tasks that assess how domain-general cognitive functions can influence linguistic task performance.

Secondly, our results provide additional evidence for the involvement of inhibitory control in language production [6,42]. This supports theories that assume word production is competitive in nature and requires inhibition to take place between activated candidates [14]. Results from individual differences studies suggest a differentiation between selective and nonselective inhibition during speech production [6,12]. However, this suggestion follows from the lack of a relation between two separate inhibition tasks, and can therefore not provide conclusive evidence that selective and nonselective inhibition are fully independent. Our results propose that both types could make use of the same inhibitory resources, at least in part [31,32]. Future research is needed in order to explore those relations in more detail in order to develop accurate models of inhibition in speech production. Finally, our results might have important theoretical implications for studying the inhibitory mechanisms underlying a number of different language disorders, such as specific language impairment [53,54], Tourette syndrome [55,56], aphasia [57], and developmental stuttering [58]. In these disorders, inhibition appears to play an important role, but it remains unclear which type of inhibition is affected.

## Conclusion

The present study provides behavioral evidence for the interplay between selective and nonselective inhibition during single word production. Combining a picture-word interference task (thought to tap selective inhibition) with a stop-signal task (nonselective inhibition), we found longer naming latencies for pictures with semantically related distractor than with unrelated distractors (semantic interference effect). In addition, we observed longer stopping latencies in the semantically related condition than in the unrelated condition, indicating that nonselective inhibition ability is influenced by selective inhibition. The results suggest that both types of inhibition share a common inhibitory mechanism, at least in part. Our findings have important theoretical implications for understanding the role of inhibition in language production.

## Supporting information

S1 Table Target names of pictures and semantically related and unrelated distractors (English translations in parentheses). (DOCX)
