# Supplementary material for: The interplay between selective and nonselective inhibition during single word production
Source: PLoS One. 2018 May 10;13(5):e0197313. doi: 10.1371/journal.pone.0197313 (PMC5945011; doi:10.1371/journal.pone.0197313)
Supplement: S1 Table — (DOCX) [file pone.0197313.s001.docx]

**S1 Table. Target names of pictures and semantically related and unrelated distractors (English translations in parentheses)**

| Target names | Related distractors | Unrelated distractors |
| --- | --- | --- |
| **Practice** |  |  |
| kruk (stool) | stoel (chair) | bij (bee) |
| mug (mosquito) | bij (bee) | speeltje (toy) |
| rits (zipper) | knoop (button) | mes (knife) |
| zwaard (sword) | mes (knife) | jetski (jet ski) |
| **Experiment** |  |  |
| bad (bathtub) | douche (shower) | tijdschrift (magazine) |
| bank(couch) | tafel (table) | worst (sausage) |
| been(leg) | arm (arm) | tafel (table) |
| boek(book) | tijdschrift (magazine) | arm (arm) |
| bom (tree) | granaat (granate) | mantel (cloak) |
| boom (bread) | plant (plant) | wanten (mittens) |
| brood (bread) | taart (cake) | knie (knee) |
| bril (glasses) | lens (lens) | plant (plant) |
| dak (roof) | tuinhuis (garden shed) | brommer (moped) |
| deur (door) | raam (window) | kaars (candle) |
| doos (box) | box (box) | vogel (bird) |
| eend (duck) | vogel (bird) | box (box) |
| ei (egg) | kip (chicken) | lens (lens) |
| fiets (bicycle) | brommer (moped) | haai (shark) |
| fles (bottle) | beker (cup) | kip (chicken) |
| hond (dog) | kat (cat) | raam (window) |
| jas (coat) | wanten (mittens) | beker (cup) |
| jurk (dress) | mantel (cloak) | tuinhuis (garden shed) |
| kaas (cheese) | worst (sausage) | teddybeer (teddy bear) |
| kam (comb) | haarspeld (hair pin) | moskee (mosque) |
| kerk (church) | moskee (mosque) | geit (goat) |
| koe (cow) | geit (goat) | planeet (planet) |
| kroon (crown) | staf (scepter) | ezel (donkey) |
| lamp (lamp) | kaars (candle) | mond (mouth) |
| maan (moon) | planeet (planet) | hoed (hat) |
| muis (mouse) | rat (rat) | haarspeld (hair pin) |
| naald (needle) | borduren (embroider) | tulp (tulip) |
| neus (nose) | mond (mouth) | borduren (embroider) |
| paard (horse) | ezel (donkey) | bus (bus) |
| pet (cap) | hoed (hat) | staf (scepter) |
| pijp (pipe) | sigaret (sigaret) | kat (cat) |
| pop (doll) | teddybeer (teddy bear) | rat (rat) |
| ring (ring) | armband (bracelet) | camper (camper) |
| roos (rose) | tulp (tulip) | granaat (granate) |
| tent (tent) | camper (camper) | armband (bracelet) |
| trein (train) | bus (bus) | doek (cloth) |
| vaas (vase) | pot (pot) | douche (shower) |
| vis (fish) | haai (shark) | sigaret (sigaret) |
| vlag (flag) | doek (cloth) | pot (pot) |
| voet (foot) | knie (knee) | taart (cake) |
